# TRPC1-mediated Ca^2+^ entry is essential for the regulation of hypoxia and nutrient depletion-dependent autophagy

**DOI:** 10.1038/cddis.2015.7

**Published:** 2015-03-05

**Authors:** P Sukumaran, Y Sun, M Vyas, B B Singh

**Affiliations:** 1 Department of Basic Sciences, School of Medicine and Health Sciences, University of North Dakota, Grand Forks, ND 58201, USA

## Abstract

Autophagy is a cellular catabolic process needed for the degradation and recycling of protein aggregates and damaged organelles. Although Ca^2+^ is suggested to have an important role in cell survival, the ion channel(s) involved in autophagy have not been identified. Here we demonstrate that increase in intracellular Ca^2+^ via transient receptor potential canonical channel-1 (TRPC1) regulates autophagy, thereby preventing cell death in two morphologically distinct cells lines. The addition of DMOG or DFO, a cell permeable hypoxia-mimetic agents, or serum starvation, induces autophagy in both epithelial and neuronal cells. The induction of autophagy increases Ca^2+^ entry via the TRPC1 channel, which was inhibited by the addition of 2APB and SKF96365. Importantly, TRPC1-mediated Ca^2+^ entry resulted in increased expression of autophagic markers that prevented cell death. Furthermore, hypoxia-mediated autophagy also increased TRPC1, but not STIM1 or Orai1, expression. Silencing of TRPC1 or inhibition of autophagy by 3-methyladenine, but not TRPC3, attenuated hypoxia-induced increase in intracellular Ca^2+^ influx, decreased autophagy, and increased cell death. Furthermore, the primary salivary gland cells isolated from mice exposed to hypoxic conditions also showed increased expression of TRPC1 as well as increase in Ca^2+^ entry along with increased expression of autophagic markers. Altogether, we provide evidence for the involvement of Ca^2+^ influx via TRPC1 in regulating autophagy to protect against cell death.

Autophagy is a cellular process responsible for the delivery of proteins or organelles to lysosomes for its degradation. Autophagy participates not only in maintaining cellular homeostasis, but also promotes cell survival during cellular stress situations.^1, 2^ The stress conditions including nutrient starvation, hypoxia conditions, invading microbes, and tumor formation, have been shown to induce autophagy that allows cell survival in these stressful or pathological situations.^1^ In addition, autophagy also recycles existing cytoplasmic components to generate the molecules that are required to sustain the most vital cellular functions.^3^ Till date, three forms of autophagy have been identified, which are designated as chaperone-mediated autophagy, microautophagy, and macroautophagy.^4^ Although the precise mechanism as to how autophagy is initiated is not well understood, many of the genes first identified in yeast that are involved in autophagy have orthologs in other eukaryotes including human homologs.^5, 6^ The presence of similar genes in all organisms suggests that autophagy might be a phenomenon that is evolutionally conserved that is essential for cell survival. In addition, since autophagy delivers a fresh pool of amino acids and other essential molecules to the cell, initiation of autophagy is highly beneficial particularly during nutritional stress situations or tissue remodeling during development and embryogenesis.^6^ Consequently, impaired or altered autophagy is often implicated in several pathologies, like neurodegenerative disorders and cancer,^7, 8, 9^ which again highlight its importance.

Ca^2+^ has a vital role in the regulation of a large number of cellular processes such as cell proliferation, survival, migration, invasion, motility, and apoptosis.^10, 11^ To perform functions on such a broad spectrum, the cells have evolved multiple mechanisms regulating cellular Ca^2+^ levels, mainly by regulating the function of various Ca^2+^ channels present in different locations. Mitochondrial, ER, lysosomal, and cytosolic Ca^2+^ levels are regulated by Ca^2+^ permeable ion channels localized either on the membranes of the intracellular organelles or on the plasma membrane.^10^ The Ca^2+^ permeable channels, including families of TRPCs, Orais, voltage-gated, two-pore, mitochondrial Ca^2+^ uniporter, IP_3_, and ryanodine receptors have all been identified to contribute towards changes in intracellular Ca^2+^ ([Ca^2+^]_i_).^10, 12, 13, 14^ Channels of the TRPCs and Orai families have been related to several Ca^2+^-dependent physiological processes in various cell types, ranging from cell proliferation to contractility, to apoptosis under both physiological and pathological conditions.^12^ Moreover, it has been suggested that intracellular Ca^2+^ is one of the key regulators of autophagy;^15^ however, the possible role of Ca^2+^ in autophagy is still inconclusive. Many reports also suggest that Ca^2+^ inhibits autophagy,^16, 17, 18^ whereas others have indicated a stimulatory role for Ca^2+^ towards autophagy.^19, 20, 21^ Furthermore, the identity of the major Ca^2+^ channel(s) involved in autophagy is not known. Members of the TRPC family have been suggested as mediators of Ca^2+^ entry into cells. Activation of the G-protein (G_q/11_–PLC pathway) leads to the generation of second messenger IP_3_.^10, 22^ IP_3_ binds to the IP_3_R, which initiates Ca^2+^ release from the ER stores, thereby facilitating stromal interacting molecule-1 (STIM1) to rearrange and activate Ca^2+^ entry via the store-operated channels.^22^ Two families of proteins (TRPCs and Orais) have been identified as potential candidates for SOC-mediated Ca^2+^ entry.^12, 22^ However, their role in autophagy has not yet been determined. Thus, here we investigated the role of Ca^2+^ entry channels (TRPCs and Orais) in autophagy and show that both hypoxia-mimetic and nutrient depression induces autophagy in two different cell lines. Furthermore, our data indicates that autophagy was dependent on TRPC1-mediated increase in intracellular Ca^2+^ levels, suggesting that TRPC1 has an important role in regulating autophagy and inhibiting cell death.

## Results

### Hypoxic conditions and serum depletion induces autophagy in both epithelial and neuronal cells

**Figure 1:**
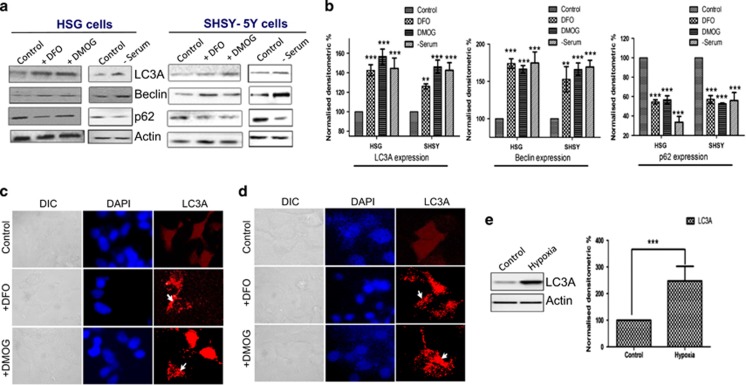
Induced Autophagy in SH-SY5Y and HSG cells. (**a**) HSG and SHSY-5Y cells were treated for 24 h with 1 mM DFO, 1 mM DMOG, and in a serum-free media. Protein was isolated and western blots represent the protein expression of different autophagy marker beclin-1, LC3A, p62, and loading control actin. (**b**) Corresponding densitometric reading of the autophagy marker protein is shown as a bar diagram. Each bar gives the mean±S.E.M. of four separate experiments. *P<0.05, **P<0.01, and ***P<0.001. Confocal image of HSG and SH-SY5Y cells, respectively, transfected with fluorescent-tagged LC3 and treated for 24 h with 200*μ*M DFO or 200*μ*M DMOG. (**c** and **d**) Western blot images showing the expression of autophagy marker LC3A in primary salivary gland cells isolated from normoxia- and hypoxia-induced mice models. (**e**) Bar diagram representing the densitometric reading of the LC3A in the above-mentioned western blots. Each bar gives the mean±S.E.M. of four separate experiments

The human salivary gland (HSG) cells and neuroblastic SHSY-5Y cells were treated with 1 mM DMOG or DFO or were subjected to serum-free media. After treatment, the cells were lysed and expressions of the autophagic marker proteins were analyzed using western blotting. Both autophagic markers Beclin-1 and LC3A were upregulated in both cells treated with DMOG or DFO or in serum-deprived conditions (Figure 1a, quantification provided in Figure 1b). Similarly, ATG5 levels were also increased under these three conditions in both HSG and SHSY-5Y cells (data not shown). Importantly, it has been previously reported that p62 expression is lost when cells undergo autophagy.^23^ Thus, we also evaluated the expression of p62, which was significantly decreased in DMOG, DFO, and serum-deprived conditions (Figures 1a and b). To have further evidence, we also evaluated the localization of LC3A as it has been previously reported that LC3B forms a punctate pattern when cells induce autophagy.^24^ Importantly, the confocal images of cells transfected with fluorescent-tagged LC3B and treated with DMOG or DFO for 24 h, confirm the result that DMOG and DFO treatment in both HSG and SHSY-5Y cells induces autophagy (Figures 1c and d). Although both DMOG and DFO could induce hypoxia, they do not truly represent hypoxic conditions. In addition, to study the physiological responses in animal models, mice were exposed to 10% oxygen for 3 days. Protein were isolated from salivary glands of normoxia- (control) and hypoxia-induced mice, as mentioned previously.^25^ Similar to our cell culture results, LC3A expression was increased in mice exhibiting hypoxia conditions (Figure 1e, quantification provided as bar graph) suggesting that hypoxia induces autophagy in mouse models.

**Figure 2:**
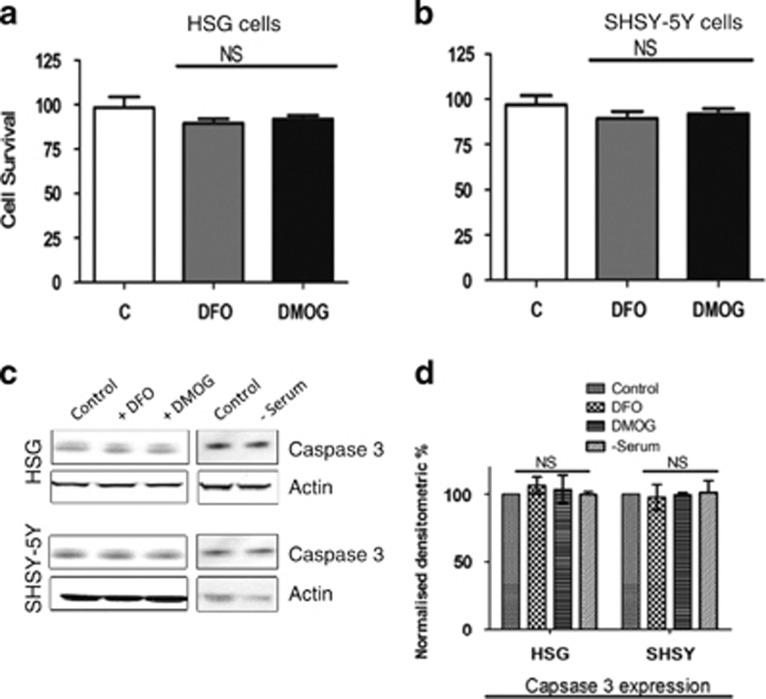
DMOG and DFO treatment effects on the cell viability or apoptosis in the cells. (**a**) Bar diagram showing the cell viability assay (MTT assay) in the HSG cells and (**b**) SHSY-5Y cells pretreated with 1 mM of both DFO and DMOG. Each bar gives the mean±S.E.M. of four separate experiments. NS indicates no significance. (**c**) Western blot images showing the expression of caspase 3 in SHSY-5Y and HSG cells pretreated with 1 mM DMOG and 1 mM DFO or in serum-free media for 24 h. (**d**) Bar diagram representing the densitometric reading of the caspase 3 expression in the above-mentioned western blots. Each bar gives the mean±S.E.M. of four separate experiments. NS indicates no significance

To confirm that hypoxia-mimetic agents, DMOG and DFO, induce autophagy that inhibits apoptosis, cell viability assays were performed. Treatment with 1 mM DMOG or 1 mM DFO showed no significant decrease in cell viability in both SHSY-5Y and HSG cells (Figures 2a and b). Moreover, the expression of caspase 3, an apoptotic marker protein, was also not altered in cells treated with DMOG or DFO or serum starvation (Figures 2c and d). Overall, these results suggest that DMOG, or DFO, or serum starvation induces autophagy in epithelial and neuronal cells that maintain cell viability perhaps by inhibiting apoptotic-mediated cell death.

### Hypoxia and serum depletion induces increase in intracellular cytosolic Ca^2+^ levels

**Figure 3:**
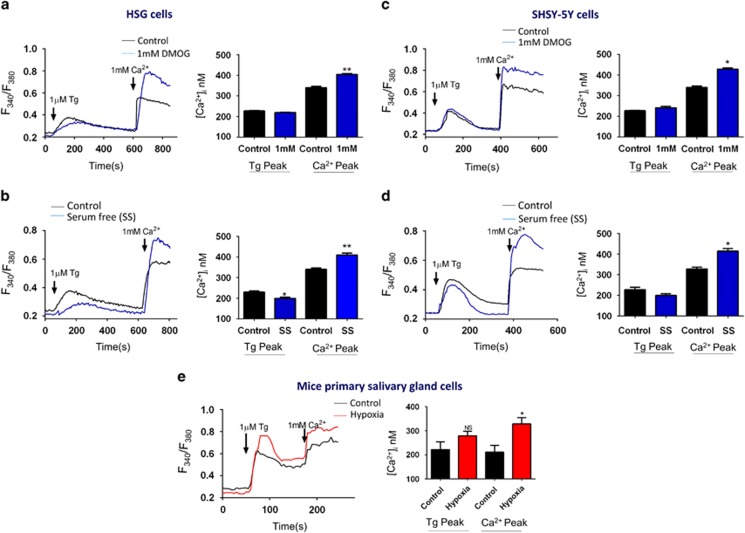
Increase intracellular calcium in autophagy induces cells. Representative traces showing the transient increase in [Ca^2+^]_i_ after addition of 1 mM calcium to HSG cells (**a**) and in SHSY-5Y cells (**b**) pretreated with 1 mM DMOG. Bar diagram shows the [Ca^2+^]_i_ in nM concentration of the above-mentioned experiment. Each bar gives the mean±S.E.M. of 50 separate experiments. *P<0.05 and **P<0.01. Representative traces showing the transient increase in [Ca^2+^]_i_ after the addition of 1 mM calcium to HSG cells (**c**) and in SHSY-5Y cells (**d**) pretreated with serum-free media. Bar diagram shows the [Ca^2+^]_i_ in nM concentration of the above-mentioned experiment. Each bar gives the mean±S.E.M. of 45 separate experiments. *P<0.05 and **P<0.01. (**e**) Representative traces showing the transient increase in [Ca^2+^]_i_ after addition of 1 mM calcium in primary salivary gland cells isolated from the hypoxia induce mice model when compared to the control (normoxia) samples. Bar diagram shows the [Ca^2+^]_i_ in nM concentration of the above-mentioned experiment. Each bar gives the mean±S.E.M. of 20 separate cells. *P<0.05

Agonist-stimulated Ca^2+^ entry has been suggested to have an essential role in regulating cell survival.^26^ Furthermore, alterations in intracellular Ca^2+^ levels has been shown to regulate autophagy.^15^ Thus, we next evaluated Ca^2+^ levels in both epithelial and neuronal cells. Cells were pretreated with 1 mM DMOG or 1 mM DFO or in serum-free media for 6 h and were then incubated with Fura-2 to measure intracellular Ca^2+^ levels in both SHSY-5Y and HSG cells. To evaluate Ca^2+^ entry, ER Ca^2+^ stores were depleted by the addition of thapsigargin (Tg, 2 *μ*M). Importantly, in the absence of extracellular Ca^2+^, the increase in [Ca^2+^]_i_ evoked by Tg (first peak) was not significantly different in DMOG-treated cells, when compared with untreated HSG cells (Figure 3a). In contrast, addition of external Ca^2+^ (1 mM), which initiates store-mediated Ca^2+^ entry, was significantly increased in DMOG-treated HSG cells (Figure 3a). Similar results were also obtained with serum starvation where a significant increase in Ca^2+^ entry was observed in HSG cells that were serum starved (Figure 3b). Consistent with these results, DFO also showed a significant increase in Ca^2+^ entry (data not shown). We next compared if these effects are same in SHSY-5Y cells, and all hypoxic-inducing treatments again resulted in an increase in [Ca^2+^]_i_ activated by store depletion in SHSY-5Y (Figures 3c and d) cells. Primary salivary gland cells were isolated from control and hypoxia-treated mice, as mentioned previously^27^ and [Ca^2+^]_i_ levels were evaluated. Consistent with cell culture results, an increase in [Ca^2+^]_i_ was again observed in cells isolated from salivary glands of hypoxia-induced mice (Figure 3e), when compared with cells from control mice. Altogether, these results suggest that conditions that induce autophagy increase [Ca^2+^]_i_ levels that might be essential for cell survival and in the induction of autophagy.

### Inhibition of TRPC channel attenuated DMOG-induced increase in intracellular Ca^2+^ levels and decreased cell viability by inducing apoptosis

**Figure 4:**
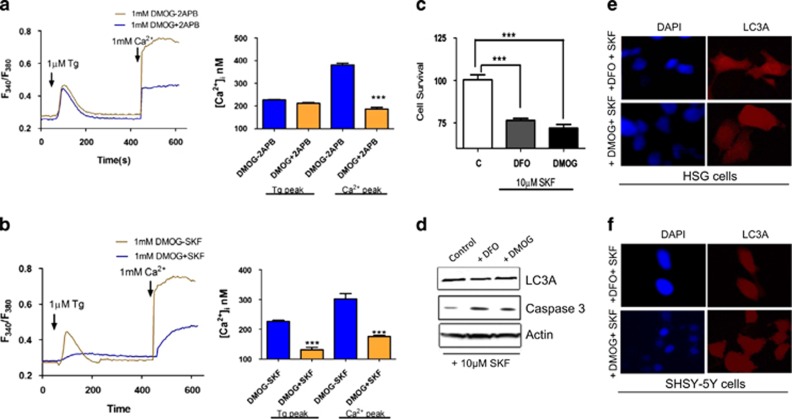
TRPC channel inhibitors attenuate the increase intracellular calcium in autophagy induces cells. (**a**) Representative traces showing the transient increase in [Ca^2+^]_i_ after the addition of 1 mM calcium in the presence of 50 *μ*M 2APB to SHSY-5Y cells pretreated with 1 mM DMOG. Bar diagram shows the [Ca^2+^]_i_ in nM concentration of the above-mentioned experiment. Each bar gives the mean±S.E.M. of 50 separate experiments. e **P<0.01 and ***P<0.001. (**b**) Representative traces showing the transient increase in [Ca^2+^]_i_ after the addition of 1 mM calcium in the presence of 10 *μ*M SKF to SHSY-5Y cells pretreated with 1 mM DMOG. Bar diagram shows the [Ca^2+^]_i_ in nM concentration of the above-mentioned experiment. Each bar gives the mean±S.E.M. of 50 separate experiments. **P<0.01 and ***P<0.001. (**c**) Bar diagram showing the cell viability assay (MTT assay) in the SHSY-5Y cells, pretreated with 1 mM DMOG in the presence of 10 *μ*M SKF. Each bar gives the mean±S.E.M. of four separate experiments. **P<0.01 and ***P<0.001. (**d**) Western blot images showing the expression of LC3A, caspase 3 in SHSY-5Y cells pretreated with 1 mM DMOG and 10 *μ*M SKF. Confocal image of HSG (**e**) and SH-SY5Y (**f**) cells transfected with GFP-LC3 and treated with 200 *μ*M DFO or 200 *μ*M DMOG and 10 *μ*M SKF

2-Aminoethoxydiphenyl borate (2APB) and SKF96365 hydrochloride (SKF) are potent TRPC channel inhibitors;^12^ we thus investigated their role in autophagy. Importantly, in both SHSY-5Y and HSG cells, the increase in [Ca^2+^]_i_ observed with DMOG treatment was attenuated in the presence of 2APB (Figure 4a) and SKF (Figure 4b). Similar results were also obtained with DFO or serum starvation where hypoxic-mediated increase in Ca^2+^ entry was decreased in cells treated with 2APB or SKF (data not shown). The cell viability was also affected when cells were pretreated with DMOG or DFO in the presence of TRPC channel inhibitor SKF, where a significant decrease in cell survival was observed (Figure 4c). Pretreatment of HSG cells with 1 mM DMOG in the presence of SKF, also resulted in a loss of autophagy, where no increase in LC3A expression was observed (Figure 4d). Moreover, an increase in apoptosis marker caspase 3 was observed in HSG cells pretreated with DMOG and TRPC channel blocker SKF (Figure 4d). Similar results were also obtained with DFO and in SHSY-5Y cells (data not shown). To further confirm this, LC3A punctate formation was evaluated in both HSG and SHSY-5Y cells. As shown in Figures 4e and f, no puncta formation of LC3B was observed in cells treated with DMOG or DFO along with SKF96365, further indicating that calcium entry via TRPC channels is essential for the induction of autophagy and inhibition of apoptosis.

### Hypoxia and serum depletion increases TRPC1 expression and Ca^2+^ entry

**Figure 5:**
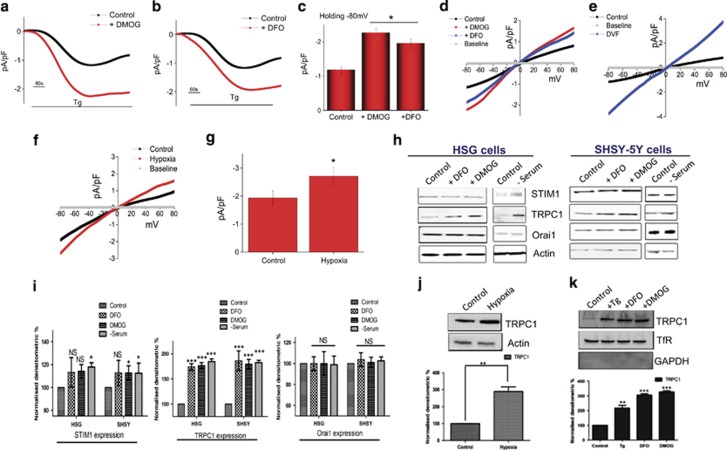
Increases in TRPC1 expression and currents in autophagy-induced HSG and SHSY-5Y cells. (**a** and **b**) Application of 1 *μ*M Tg in bath solution induced inward currents at −80 mV in control, 1 mM DMOG-, and 1 mM DFO-treated cells. (**c** and **d**) Respectively *I*–*V* curves under these conditions are shown in **c** and quantitation (8–10 recordings) of current intensity at −80 mV is shown in **d**, *P=<0.05. (**e**) *I*–*V* curves of currents induced by the application of 1 *μ*M Tg in standard external Ringer's solution and Na-based DVF external solutions. (**f**) Bath application of 1 *μ*M Tg in bath solution induced in salivary gland cells and relative *I*–*V* curves. (**g**) Average (8–10 recordings) current intensity at −80 mV under these conditions is shown, *P=<0.05. (**h**) Represents western blot images showing the expression of SOCE components, STIM1, Orai1, and TRPC1 in HSG and SHSY-5Y cells pretreated with 1 mM DMOG and 1 mM DFO for 24 h. Corresponding densitometric reading of the protein is shown as a bar diagram (**i**). Each bar gives the mean±S.E.M. of four separate experiments. *P<0.05, **P<0.01, and ***P<0.001. (**j**) Western blot images showing the expression of TRPC1 in primary salivary gland cells isolated from control and hypoxia-induced mice models. Bar diagram representing the densitometric reading of the TRPC1 in the above-mentioned western blots. Each bar gives the mean±S.E.M. of four separate experiments. (**k**) Western blot images showing the relative surface expression of TRPC1 obtained from cell surface biotinylation. Bar diagram shows the normalized expression of TRPC1 to the expression of cell surface transferrin receptor (TfR) protein. Each bar gives the mean±S.E.M. of three separate experiments

To establish the identity of the Ca^2+^ entry channel, electrophysiological recordings were performed in hypoxia conditions. The addition of Tg caused the appearance of an inward current, which reversed between 0 and −5 mV in SHSY-5Y cells (Figures 5a–d) and in HSG cells (results not shown). Perfusion with Na-based DVF solution facilitated the current, which indicated the current is nonselective (Figure 5e). The channel properties were similar to those previously observed with TRPC1 channels,^28^ which was induced by store deletion (baseline currents without Tg are shown in *I*–*V* curves), reversal potential around 0 mV, slightly inward rectifying and nonselective, suggesting that TRPC1 could contribute to the endogenous Ca^2+^ entry channel in these cells. Importantly, DMOG and DFO treatment significantly facilitated TRPC1-mediated Ca^2+^ currents without altering the current–voltage (*I*–*V*) relationship (Figure 5a–d). Moreover, electrophysiological recordings using dispersed salivary gland cells also showed an inward nonselective current upon addition of Tg, which are consistent with previous results.^27, 29^ The properties of the current are similar to TRPC1 current and more importantly, hypoxia-treated cells significantly facilitated the Tg-mediated I_soc_-currents (Figures 5f and g). To further identify the cellular component of Ca^2+^ entry channel(s) involved in this process, cell lysates were obtained under these conditions and were used to evaluate the expression of various proteins involved in Ca^2+^ entry. Importantly, cells pretreated with 1 mM DMOG, or 1 mM DFO, or in serum-free media showed a significant increase in TRPC1 levels (Figure 5h). In contrast, no significant change in either STIM1 or Orai1 expression levels were observed in hypoxia or serum-depletion conditions in both SHSY-5Y and HSG cells (Figures 5h and i). TRPC1 expression was also increased in hypoxia-induced mice models (Figure 5j). To further establish as to how hypoxia-increased calcium entry surface expression of TRPC1 was observed in SHSY-5Y cells, cells were pretreated with 1 mM DMOG or 1 mM DFO. As indicated in Figure 5k, cells treated with DFO or DMOG showed increase in surface expression of TRPC1, but not transferrin receptor (as internal control), which could account for the increase in hypoxia-mediated Ca^2+^ currents observed above. Consistent with previous reports,^30^ addition of thapsigargin also induced surface expression of TRPC1 (as positive control). Hereby, our data indicate that autophagy was dependent on TRPC1, suggesting that TRPC1 has an important role in regulating hypoxia inducing autophagy and inhibiting cell death.

### Knockdown of TRPC1, but not TRPC3, or inhibition of autophagy attenuated DMOG and serum depletion induced increase in intracellular Ca^2+^ and affects cell viability

**Figure 6:**
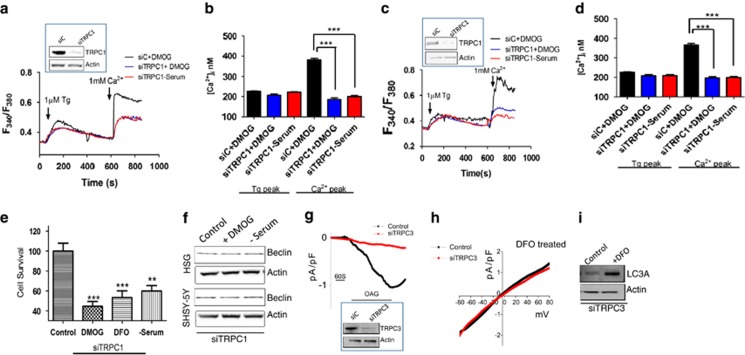
Knockdown of TRPC1 attenuated the autophagy-induced intracellular calcium increase and also affects the cell viability. Western blot images showing the knockdown of TRPC1 using siRNA, HSG cells (**a**) (70% knockdown, *n*=3, *P*<0.01), and SHSY-5Y cells (**b**) (72% knockdown, *n*=3, p<0.001). Representative traces showing the transient increase in [Ca^2+^]_i_ after the addition of 1 mM calcium to siRNA TRPC1 knockdown HSG cells (**a**) and in SHSY-5Y cells (**b**) pretreated with 1 mM DMOG or in serum-free media. Bar diagram (**c** and **d**) shows the [Ca^2+^]_i_ in nM concentration of the above-mentioned experiment. Each bar gives the mean±S.E.M. of 40 separate cells. *P<0.05 and ***P<0.001. (**e**) Bar diagram showing the cell viability assay (MTT assay) in the TRPC1 knockdown SHSY-5Y cells, pretreated with 1 mM DMOG or 1 mM DFO or in serum-free media. Each bar gives the mean±S.E.M. of four separate experiments. **P<0.01. (**f**) Western blot images showing the expression of autophagy marker beclin-1 in HSG and SHSY-5Y cells pretreated with 1 mM DMOG or in serum-free media for 24 h. (**g**) Western blot image showing the TRPC3 knockdown using siRNA in SHSY-5Y cells (60% knockdown, *n*=3, *P*<0.01). Application of 50 *μ*M OAG in bath solution induced inward currents at −80 mV in control and TRPC3 knockout cells. (**h**) Under DFO treatment, respectively *I*–*V* curves of currents induced by the application of 1 *μ*M Tg in control and TRPC3 knockout cells treated with 1 mM DFO. The traces are representative of average (8–10 recordings) of current intensity at −80 mV. (**i**) Western blot images showing the expression of LC3A in siTRPC3 SHSY-5Y cells with and without 24 h pretreatment with 1 mM DFO (*n*=3, *P*<0.05)

Data presented thus far indicate that TRPC1 could be important for hypoxia- and serum starvation induced increase in Ca^2+^ entry and could modulate autophagy. Thus, to study the importance of TRPC1 channels in hypoxia and serum depletion-induced autophagy, we knocked down TRPC1 in both SHSY-5Y and HSG cells using siRNA (70% knockdown Figures 6a and c shown as insets). Importantly, the DMOG- and serum depletion-induced increase in Ca^2+^ entry was attenuated in TRPC1 knockdown cells in both SHSY-5Y and HSG cells (Figures 6a–d). Moreover, pretreatment of DMOG, or DFO or serum-free media in TRPC1 knockdown cells also showed an increase in apoptosis (Figure 6e) and autophagy was inhibited as observed by beclin-1 levels (Figure 6f). To further position TRPC1 as the main calcium channel responsible for the currents induced by hypoxia, we knocked down TRPC3 in these cells. We first investigated the TRPC3 channel activity, which was induced by the application of OAG and addition of OAG induced an inward current, which was abolished by the expression of siTRPC3 (Figure 6g). More importantly, siTRPC3 has no significant effect on hypoxia- (1 mM DFO treatment) induced increase in Tg-induced currents (Figure 6h) and LC3A expression (Figure 6i). Collectively, these results suggest that hypoxia and serum starvation-mediated increase in Ca^2+^ entry is at least in part mediated via the TRPC1 channel, which could lead to the activation of autophagy that together would inhibit cell death in SHSY-5Y and HSG cells.

**Figure 7:**
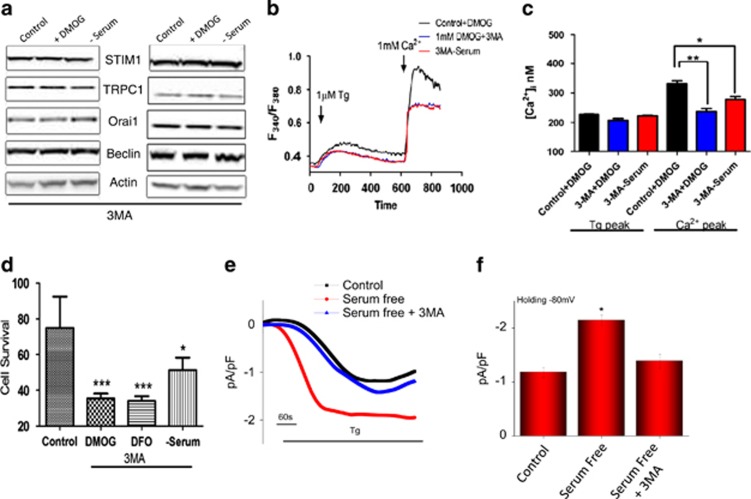
Pretreatment with autophagy inhibitor 3-methyladenine (3-MA) attenuated the intracellular calcium influx and induces apoptosis. (**a**) Western blot images showing the expression of SOCE components STIM1, TRPC1, and Orai1, autophagy marker beclin-1 and loading control actin in HSG and SHSY-5Y cells pretreated with 1 mM DMOG or in serum-free media in the presence of 1 mM autophagy marker 3-MA for 24 h. (**b**) Representative traces showing the transient increase in [Ca^2+^]_i_ after the addition of 1 mM calcium in the presence of 1 mM 3-MA to SHSY-5Y cells pretreated with 1 mM DMOG or in serum-free media. (**c**) Bar diagram shows the [Ca^2+^]_i_ in nM concentration of the above-mentioned experiment. Each bar gives the mean±S.E.M. of 50 separate experiments. *P<0.05, **P<0.01. (**d**) Bar diagram showing the cell viability assay (MTT assay) in the SH-SY5Y cells, pretreated with 1 mM DMOG in the presence of 1 mM. Each bar gives the mean±S.E.M. of four separate experiments. ***P<0.001. (**e**) Application of 1 *μ*M Tg in bath solution induced inward currents at −80 mV in control, cells treated in serum-free media and autophagy inhibitor 3-MA-treated cells. (**f**) Average (8–10) recordings current intensity at −80 mV are shown, *P<0.05

To further understand the link between Ca^2+^ entry via the TRPC1 channel and autophagy, 3-methyladenine (3-MA) a known autophagy inhibitor,^4^ was used. The cells were pretreated with 1 mM 3-MA, along with DMOG, or DFO, or serum-free media and protein expression of STIM1, Orai1, TRPC1, and autophagy markers were analyzed. Importantly, cells treated with 3-MA showed no increase in DMOG or serum-deprived increase in TRPC1 expression (Figure 7a). Furthermore, no increase in beclin-1 an autophagic marker was observed in cells pretreated with 3-MA along with DMOG- or serum-deprived conditions (Figure 7a). Pretreatment with 3-MA also attenuated the increase in Ca^2+^ entry that was induced by DMOG and serum starvation in both SHSY-5Y (Figures 7b and c) and HSG cells (result not shown). Consistent with these results, pretreatment with 3-MA also attenuated cell viability (Figure 7d) and serum starvation induced increase in Tg-induced Ca^2+^ currents (Figures 7e and f). Altogether, these data suggest that Ca^2+^ entry via the TRPC1 channel is essential for autophagy that leads to the inhibition of cell death and loss of TRPC1 function or autophagy could lead to decrease in cell survival.

## Discussion

When cells encounter stressful situations, like nutrient depletion or hypoxia they either try to survive under these conditions by coping with the stress, or can activate a programmed cell death mechanism such as apoptosis.^2^ One of the most beneficial processes during stress adaptation is the stimulation of autophagy, a lysosomal delivery pathway.^6^ Autophagy is also a major pathway needed for the clearance of pathogens in immunity and helps in maintaining cellular homeostasis.^15^ In 1993, Gordon *et al.*^31^ first reported on Ca^2+^-dependent regulation of autophagy and suggested a complex role for Ca^2+^, as both chelation of either intra- and extracellular Ca^2+^ as well as elevating cytosolic [Ca^2+^]_i_ levels suppressed autophagy. This dichotomy could be due to the amount of increase in Ca^2+^ as well as the source of Ca^2+^ entry. Our data suggest that a slight increase in Ca^2+^ entry was actually protective under these stressful conditions. Importantly, Ca^2+^ has also been shown to promote cell proliferation as well as induce apoptosis,^32, 33, 34^ suggesting that Ca^2+^ entry through different channels might dictate the fate of the cell differently. Consistent with this notion, treatments with AMPA or KCl have shown to increase [Ca^2+^]_i_ without causing toxicity,^35, 36^ whereas equally high Ca^2+^ loads were shown to be toxic when Ca^2+^ enters via the NMDA channels.^37^ Furthermore, in some cells, decreasing [Ca^2+^]_i_ could be toxic, whereas in others, modest increase in [Ca^2+^]_i_ can be protective, indicating a ‘set-point' mechanism for the effect of [Ca^2+^]_i_ in various cells.^12, 26, 38, 39^

The notion that Ca^2+^ is important for cell survival is not new, but only recently has this topic regained interest. However, the published results are again conflicting with regard to the role of intracellular Ca^2+^ in autophagy. Many reports suggest that Ca^2+^ and Ca^2+^-handling proteins inhibit autophagy,^16, 17, 18^ whereas other reports indicate a stimulatory role for Ca^2+^ toward autophagy.^19, 20, 21^ Our data support the idea that Ca^2+^ entry via TRPC1 is essential for the induction of autophagy. Ca^2+^-mediated inhibition of autophagy has been mostly focused on inositol 1,4,5, triphosphate (IP_3_) receptor (IP_3_R).^15, 16^ In Huntington's disease cell model, lithium ions have been shown to stimulate autophagy in a novel mTOR-independent manner.^40^ Lithium acted through inhibition of the inositol monophosphatases, thereby reducing the IP_3_ levels, which reduced the stimulatory effect of lithium on autophagy. This is consistent with the IP_3_R triple knockout chicken DT40 cells, which showed higher autophagy levels than their wild-type counterparts, verifying the IP_3_R-mediated inhibition of autophagy.^18^ Autophagy has also been shown to be initiated by chemical inhibition of IP_3_Rs or suppressing its expression thereby suppressing the Ca^2+^ signaling.^41^ This is consistent with our data as loss of IP_3_R will have a decrease in TRPC1-mediated Ca^2+^ entry. Although we have not fully identified the mechanism as to how TRPC1 regulates autophagy, it has been shown that IP_3_Rs facilitate the formation of anti-autophagic Bcl-2–Beclin-1 complex thereby decreasing the amount of free Beclin-1 available to induce autophagy. We suggest that perhaps similar mechanism might be present where binding of IP_3_R with TRPC1 will inhibit the formation of anti-autophagic complex and more research is needed to understand the mechanism via which TRPC1 regulates autophagy. Binding of xestospongin-B to the IP_3_R also induced the dissociation of the complex, which was prevented by Bcl-2 overexpression, whereas siRNA against Bcl-2 abolished the IP_3_R–Beclin-1 interaction.^17^

Ca^2+^ mobilizing agents, like ionomycin, ATP, vitamin D3, sarco/endoplasmic reticulum Ca^2+^ ATPase (SERCA) inhibitors thapsigargin and alisol have been reported to act as an activator of autophagy by elevating [Ca^2+^]_i_.^7, 19, 39, 42, 43^ Our data not only support these studies, but also identify that Ca^2+^ entry via TRPC1 is essential for autophagy. Of course, prolonged treatment with these agents will lead to ER Ca^2+^ depletion and subsequent ER stress, which itself might also be a trigger for autophagy. The addition of BAPTA-AM, a fast and potent intracellular Ca^2+^ buffer, however, prevented the induction of autophagy, indicating the importance of cytosolic Ca^2+^.^44^ The thapsigargin-induced autophagy was still present in unfolded protein response (UPR)-deficient cells, suggesting again a direct involvement of Ca^2+^ in the induction of autophagy.^45^ The nutrient deprivation-induced autophagy was also inhibited by BAPTA-AM,^46^ suggesting that other autophagy inducers (like starvation, rapamycin, etc.) might also lead via enhanced Ca^2+^ signaling to autophagy induction,^15^ which is again consistent with the data presented here. Although our data suggest that TRPC1 was important for autophagy (as silencing of TRPC1, but not TRPC3, inhibited autophagy), we cannot completely rule out the possible role of other Ca^2+^ channels in this process. Gordon *et al.*^31^ already suggested the dependence of autophagy on intracellular stored Ca^2+^ and most Ca^2+^-mobilizing agents promote Ca^2+^ release from the ER.^15^ Ca^2+^-dependent autophagy is also enhanced by the pharmacological agent PK11195.^47^ Similarly, it has been shown that inhibition of plasma membrane Ca^2+^ channels prevented the autophagy induction caused by the increase of intra-axonal Ca^2+^ in the rat optic nerve after crush lesion;^48^ however, the source as well as the Ca^2+^ channels responsible for this has not been identified. These results along with the data presented here clearly show that the ER is probably the main, but not the only origin for Ca^2+^ during Ca^2+^-induced autophagy. Lowering of ER [Ca^2+^] also prevents apoptosis driven by mitochondrial Ca^2+^ overload, underscoring that both autophagy and apoptosis are closely related.^15^ Our data also suggest that Ca^2+^ entry via the TRPC1 channel was initially protective. However, most of the compounds used for Ca^2+^-induced autophagy have also been shown to promote apoptosis.^3, 7, 15, 19, 39^ Therefore, one might propose that autophagy is indirectly activated by Ca^2+^ that tries to counter apoptosis^20^ to protect these cells and loss of these functions could perhaps lead to apoptosis. Here, thus we propose a model where increase in intracellular Ca^2+^ via TRPC1 channel promotes autophagy before the activation of apoptosis and increases cell survival.

## Materials and Methods

### Cell culture, animals, reagents, transfections, and RNAi

Human salivary gland (HSG) cells and SHSY-5Y neuroblastoma cells were cultured in their respective medium along with various supplements, as previously described.^32, 49, 50^ Cells were maintained at 37 °C with 95% humidified air and 5% CO_2_ and were passaged as needed. Culture medium was changed twice weekly and cells were maintained in complete media, until reaching 90% confluence. For RNAi experiments, siRNA that targets the coding sequence of human TRPC1 or TRPC3 were obtained from Ambion (Life Technology, Carlsbad, CA, USA) and a FITC-conjugated non targeting siRNA was used as control. Cells were transfected with individual siRNA (50 nM) using Lipofectamine 2000 in Opti-MEM medium as per supplier's instructions (Invitrogen, Life Technology) and assayed after 48 h. Antibodies that were used in this study are described in the figures. All other reagents used were of molecular biology grade obtained from Sigma (St. Louis, MO, USA) chemicals unless mentioned otherwise. To subject mice to chronic hypoxia, mice were placed in a plexiglass chamber maintained at 21% (normal) or 10% O_2_ (hypoxic) by controlling the inflow rates of air and nitrogen. The O_2_ concentration was monitored continuously and after 3 days, mice were anesthetized and salivary glands were obtained and used for the study.

### Cell viability assays

Cells were seeded on 96-well plates at a density of 0.5 × 10^5^ cells/well. The cultures were grown for 24 h followed by the addition of fresh medium before the experiment. Cell viability was measured by using the MTT method. Twenty microliters of MTT reagent (5 mg/ml MTT in PBS) was added to each well and incubated in a CO_2_ incubator for 4 h. The resulting formazan dye was extracted with 100 *μ*l of 0.01 N HCl in isopropanol and the absorbance was measured in a micro plate reader (Molecular Device, Sunnyvale, CA, USA) at 570 and 650 nm. Cell viability was expressed as a percentage of the control culture.

### Cell surface biotinylation, membrane preparations, and western blot analyses

Cells were collected and stored at −80 °C. Crude lysates were prepared from SH-SY5Y, HSG cells, and salivary tissues, as described previously in Singh *et al.*^51^ and Sukumarn *et al.*^52^ Protein concentrations were determined using the Bradford reagent (Bio-Rad, Hercules, CA, USA), and 25–50 *μ*g of proteins were resolved on 4–12% SDS-Tris-acetate gels, transferred to PVDF membranes and probed with respective antibodies Peroxidase conjugated respective secondary antibodies were used to label the proteins. The proteins were detected by enhanced chemiluminescence detection kit (SuperSignal West Pico; Pierce, Thermo Scientific, Waltham, MA, USA). Densitometric analysis was performed using imageJ analysis and results were corrected for protein loading by normalization for *β*-actin expression as described in Singh *et al.*^51, 53, 54, 55^ For cell surface biotinylation, cells were treated as required and incubated for 20 min with 1.5 mg/ml Sulfo-NHS-LC-Biotin (Pierce) in PBS (pH 8.0) on ice and followed the protocol as mentioned before.^55, 56^ Following biotin labeling, cells were washed and solubilized. Biotinylated proteins were pulled down with NeutrAvidin-linked beads (Pierce), resolved on 4–12% SDS-Tris-acetate gels, and individual proteins were detected by western blotting.

### Confocal microscopy

For the immunofluorescence assays, the SH-SY5Y and HSG cells were grown overnight on coverslips. Cells were transfected with fluorescent-tagged LC3A plasmid using Lipofectamine 2000 in Opti-MEM medium as per supplier's instructions (Invitrogen) and treated with the desired chemicals. After 24 h, the cells were washed twice with phosphate-buffered saline, and fixed for 30 min using 3% paraformaldehyde. Confocal images were collected using a MRC1024-krypton/argon laser scanning confocal equipped with a Zeiss LSM 510 Meta photomicroscope (Carl Zeiss, Jena, Germany).

### Calcium measurement

Cells were incubated with 2 *μ*M fura-2 (Molecular Probes, Eugene, OR, USA) for 45 min, washed twice with Ca^2+^-free SES (Standard External Solution, include: 10 mM HEPES, 120 mM NaCl, 5.4 mM KCl, 1 mM MgCl2, 10 mM glucose, pH 7.4) buffer. For fluorescence measurements, the fluorescence intensity of Fura-2-loaded control cells was monitored with a CCD camera-based imaging system (Compix, Cranbery, PA, USA) mounted on an Olympus (Shinjuku, Tokyo, Japan) XL70 inverted microscope equipped with an Olympus × 40 (1.3 NA) objective. A monochrometer dual wavelength enabled alternative excitation at 340 and 380 nm, whereas the emission fluorescence was monitored at 510 nm with an Okra Imaging camera (Hamamatsu, Shizuoka Prefecture, Japan). The images of multiple cells collected at each excitation wavelength were processed using the C imaging, PCI software (Compix), to provide ratios of Fura-2 fluorescence from excitation at 340 nm to that from excitation at 380 nm (F340/F380). Dispersed salivary gland cells were placed on glass-bottom poly-D lysine plates and used for the study. Fluorescence traces shown represent [Ca^2+^]_i_ values that are averages from at least 30–40 cells and are a representative of results obtained in at least three to four individual experiments. [Ca^2+^]_i_ in individual cells was estimated on the basis of the Grynkiewicz equation formula: [Ca^2+^]_i_=*K*_D_ × *B* × (*R*−*R*_min_)/(*R*_max_−*R*), where *K*_D_ is the indicator's dissociation constant for Ca^2+^(0.22 *μ*M); *R* is ratio of fluorescence intensity at two different wavelengths (340/380 nm); *R*_max_ and *R*_min_ are the ratios of Ca^2+^-free and Ca^2+^-bound Fura-2, respectively; and *B* is the ratio of the fluorescence intensity of the second excitation wavelength at zero and saturating Ca^2+^ concentrations.^57^ The bar diagram shown in the figures represents the [Ca^2+^]_i_ in nanomolar concentrations.

### Electrophysiology

For patch clamp experiments, coverslips with cells were transferred to the recording chamber and perfused with an external Ringer's solution of the following composition (mM): NaCl, 145; CsCl, 5; MgCl_2_, 1; CaCl_2_, 1; Hepes, 10; Glucose, 10; pH 7.3 (NaOH). For Na-based divalent ion-free (DVF), the external solutions contained (mM) 165 NaCl, 5 CsCl, 10 EDTA, 10 HEPES and 10 glucose, pH 7.4. Whole-cell currents were recorded using an Axopatch 200B (Axon Instruments, Sunnyvale, CA, USA). The patch pipette had resistances between 3 and 5 M after filling with the standard intracellular solution that contained the following (mM): cesium methane sulfonate, 150; NaCl, 8; Hepes, 10; EGTA, 10; pH 7.2 (CsOH). With a holding potential 0 mV, voltage ramps ranging from −100 mV to +100 mV and 100 ms duration were delivered at 2-s intervals after whole-cell configuration was formed. Currents were recorded at 2 kHz and digitized at 5–8 kHz. pClamp 10.1 software was used for data acquisition and analysis. Basal leaks were subtracted from the final currents and average currents are shown. All experiments were carried out under room temperature.

### Statistics

Data analysis was performed using MicroSoft Excel or Origin 7.0 (OriginLab) and Graphpad prism 6.0. Statistical comparisons were made using one-way ANOVA. Experimental values are expressed as means±S.E.M. Differences in the mean values were considered to be significant at *P<0.05 or **P<0.01, respectively.

## Abbreviations

TRPC: transient receptor potential canonical
STIM1: stromal interaction molecule-1
ER: endoplasmic reticulum
DMOG: dimethyloxalylglycine
DFO: desferrioxamine
2APB: 2-aminoethoxydiphenyl borate
SOC: store-operated calcium entry
IP_3_R: inositol 1,4,5, triphosphate (IP_3_) receptor
Tg: thapsigargin
HSG: human salivary gland
mTOR: mammalian target of rapamycin
3-MA: 3-methyladenine
